# Patients’ perspective of barriers and facilitators to taking long-term controller medication for asthma: a novel taxonomy

**DOI:** 10.1186/s12890-015-0044-9

**Published:** 2015-04-25

**Authors:** Sandra Peláez, Alexandrine J Lamontagne, Johanne Collin, Annie Gauthier, Roland M Grad, Lucie Blais, Kim L Lavoie, Simon L Bacon, Pierre Ernst, Hélène Guay, Martha L McKinney, Francine M Ducharme

**Affiliations:** Clinical Research and Knowledge Transfer Unit on Childhood Asthma, Research Centre, Sainte-Justine University Health Centre Montreal, Quebec, Canada; Department of Pharmacology, University of Montreal, Montreal, Quebec, Canada; Department of Family Medicine, Jewish General Hospital, Montreal, Quebec, Canada; Department of Psychology, Université du Québec à Montréal, Montreal, Quebec, Canada; Department of Exercise Sciences, Concordia University, Montreal, Quebec, Canada; Department of Pulmonary Medicine, Jewish General Hospital, Montreal, Quebec, Canada; Department of Epidemiology, Biostatistics and Occupational Health, McGill University, Montreal, Quebec, Canada; Institut national d’excellence en santé et en services sociaux, Quebec, Quebec, Canada; Department of Paediatrics, University of Montreal, Montreal, Quebec, Canada; Department of Social and Preventive Medicine, University of Montreal, Montreal, Quebec, Canada; Departments of Pediatrics and of Social and Preventive Medicine, Associate Director of Clinical Research, Research Centre, CHU Sainte-Justine, 3175 Côte Sainte-Catherine, Room 7939, Montreal, Quebec, H3T 1C5 Canada

**Keywords:** Asthma, Patient perspective, Adherence, Long-term daily controller medication, Inhaled corticosteroids, Barriers, Facilitators

## Abstract

**Background:**

Although asthma morbidity can be prevented through long-term controller medication, most patients with persistent asthma do not take their daily inhaled corticosteroid. The objective of this study was to gather patients’ insights into barriers and facilitators to taking long-term daily inhaled corticosteroids as basis for future knowledge translation interventions.

**Methods:**

We conducted a collective qualitative case study. We interviewed 24 adults, adolescents, or parents of children, with asthma who had received a prescription of long-term inhaled corticosteroids in the previous year. The one-hour face-to-face interviews revolved around patients’ perceptions of asthma, use of asthma medications, current self-management, prior changes in self-management, as well as patient-physician relationship. We sought barriers and facilitators to optimal asthma management. Interviews were transcribed verbatim and transcripts were analyzed using a thematic approach.

**Results:**

Patients were aged 2–76 years old and 58% were female. Nine patients were followed by an asthma specialist (pulmonologist or allergist), 13 patients by family doctors or pediatricians, and two patients had no regular follow-up. Barriers and facilitators to long-term daily inhaled corticosteroids were classified into the following loci of responsibility and its corresponding domains: (1) patient (cognition; motivation, attitudes and preferences; practical implementation; and parental support); (2) patient-physician interaction (communication and patient-physician relationship); and (3) health care system (resources and services). Patients recognized that several barriers and facilitators fell within their own responsibility. They also underlined the crucial impact (positive or negative) on their adherence of the quality of patient-physician interaction and health care system accessibility.

**Conclusions:**

We identified a close relationship between reported barriers and facilitators to adherence to long-term daily controller medication for asthma within three loci of responsibility. As such, patients’ adherence must be approached as a multi-level phenomenon; moreover, interventions targeting the patient, the patient-physician interaction, and the health care system are recommended. The present study offers a potential taxonomy of barriers and facilitators to adherence to long-term daily inhaled corticosteroids therapy that, once validated, may be used for planning a knowledge translation intervention and may be applicable to other chronic conditions.

**Electronic supplementary material:**

The online version of this article (doi:10.1186/s12890-015-0044-9) contains supplementary material, which is available to authorized users.

## Background

Nearly 60% of patients with persistent asthma have suboptimal asthma control, a figure that has remained unchanged since 2000 in Canada and abroad [1-3]. Suboptimal asthma control is associated with preventable asthma symptoms, overuse of bronchodilators (i.e., rescue medication), functional impairment (e.g., work absenteeism), increased health service use (e.g., emergency visits), and even death [4,5]. The goal of asthma management is to control the disease so that patients may lead a normal active life and prevent long-term impairment. [6,7]. Through guided self-management plans, patients learn to manage their asthma under the recommendations of their physician [6,8,9]. The cornerstone of asthma management in individuals with persistent asthma is the daily use of long-term controller medication for the disease, most notably, inhaled corticosteroids [3]. Unfortunately, adherence to recommended medications remains very low and is recognized as the main cause of therapeutic failure and preventable asthma morbidity [10,11].

The lack of prior identification of relevant barriers and facilitators before planning an intervention is one of the main reasons why knowledge translation interventions aiming at various clinical issues have either failed or had a modest effect [12]. Whereas barriers to adherence to long-term controller medication for asthma have been extensively studied in the last decade, the literature on facilitators of adherence to asthma controller medication is scant.

Most commonly reported barriers to long-term controller medication for asthma include: fear of adverse effects, addiction, or dependence to medication; belief that the medication does not help or is not necessary; concerns about a diminishing effectiveness of medication over time; preference for alternative asthma management; sense of only an intermittent need for medication; inconvenient dosage regimens; inadequate knowledge of the medication and/or the disease; cost of the medication; stigmatization of the affected individual; dislike of the provider; medical comorbidity; and the lack of social support [13-19]. In contrast, previously identified facilitators are: simple dosage regimens (e.g., one-daily dose); patients’ knowledge related to the medication and the disease; access to high-quality personalized health care; coping with the disease; appropriate asthma self-management; and, possessing a written action plan [16,17,19-21]. A first taxonomy of facilitators of physicians’ prescription behavior in the acute care setting, which included 5 domains (knowledge, attitude, and beliefs; operationalization of guidelines; choice of medication; parent or patient-related factors; and setting-related factors), was developed [22]. However, whether these facilitators would apply to patients’ behavior regarding long-term medication intake, that is, over months and years, in the non-acute setting is uncertain.

To our knowledge, no study has explored how patients perceive the dynamic relationship between barriers to, and potential facilitators of, daily medication adherence, and no classification system of potential facilitators exists on which to survey specific groups of patients before planning a knowledge translation intervention. The objective of the present study was to gain insight into factors perceived by patients as hindering or fostering their daily adherence to inhaled corticosteroids, the preferred medication to achieve asthma control. We also aimed to develop a taxonomy of barriers and facilitators to support the development of knowledge translation interventions to increase patients’ adherence to daily controller medication for asthma over the long-term [23].

## Methods

### Design and procedures

We designed a collective qualitative case study [24]. A similar study conducted in parallel, focusing on physicians’ perspective, has been the object of a recent publication [25]. The study was approved by the Research Ethics Board of the Sainte-Justine University Health Centre; written informed consent from all participants and parents, as well as written assent from children aged 7 to 17 years, were obtained. In the informed consents the participants both agreed to participate and acknowledged that data would be used for research dissemination.

The participants were recruited through advertising in a free daily newspaper distributed in the greater Montreal area and by word of mouth. Participants were selected using criterion-based sampling [26] and were eligible if they: (1) had (or were parents of a child with) a confirmed diagnosis of asthma; (2) had visited a physician and renewed a prescription for inhaled corticosteroids in the past year, and (3) spoke and understood English or French. Children under 12 years old were interviewed with their parents. A financial compensation of CAD $50 was given to each participant.

A one-hour face-to-face interview was conducted by two experienced researchers (SP and AG) between June and September 2011. The interview revolved around patients’ perceptions of their asthma, use of asthma medications, current self-management, prior changes in self-management, and patient-physician relationship. The interview guide was developed in French and translated into English. A copy of the English version is available in Appendix A. Examples of the questions were: “Why do you take (or not) the medication in the way you have just described? What would you think/do if your doctor recommends that you take long-term controller medication (i.e., inhaled corticosteroids)?” We adhered to the idea that “knowledge is co-constructed in the interaction between the interviewer and the interviewee” [27]. Thus, although we followed the interview guide, we remained open and flexible to discuss participants’ ideas. When needed, we used *detail-oriented (e.g.,* When did that happen? Who else was involved?) and *clarification probes* (e.g., What exactly do you mean?) to foster patients’ description and illustration of discussed topics [26]. The interview was pilot-tested with a parent of a child with asthma.

### Data analysis

All interviews were audio-recorded, transcribed verbatim, and reviewed by the interviewers for accuracy [28]. For confidentiality purposes, each transcript was identified with two letters and a unique number indicating the order in which the patients were recruited.

We used thematic analysis [29] to analyze the data. Thematic analysis includes six phases: familiarizing with data (e.g., reading the data), generating initial codes (e.g., coding interesting features), searching for themes (e.g., collating codes into potential themes), reviewing themes (e.g., checking codes represent the themes), defining and naming themes (e.g., refining analysis), and producing the report. The analysis was guided by previous described barriers and facilitators, as well as the “multi-level model of asthma disparities” [30]. We chose this model because, although it was developed to explain the mechanisms involved in observed disparities in asthma, it integrates essential research evidence that helps to understand treatment non-adherence.

We conducted data analysis in parallel to ongoing data collection. The first author holistically read and coded all interviews and the fourth author (AG) coded 17 (71%) interviews. Double-coded interviews were compared and disagreements were discussed until consensus was achieved. A preliminary coding scheme was developed and discussed with two other team researchers (AJM and FMD). Once the coding scheme achieved consistency, it was discussed with the whole research team to substantiate the interpretation of results.

We used the computer software MAXQDA (VERBI GmbH, Germany, version 10) to support data analysis. To better represent patients’ words, we selected quotes from interviews recognizing that, when necessary, some of these quotes were edited with regard to grammar and syntax to enhance the clarity [28]. Back translation was used to translate interview questions and relevant participant quotes [31].

## Results

### Participants’ characteristics

We interviewed 24 individuals: 16 adults with asthma with a median (range) age of 44 (18 to 76) years, two adolescents with asthma aged 15 and 18 years, and six parents of children with asthma with a median (range) age of 8.5 (2 to 12) years, 59% of participants were female. Five of the six children were 9 to 12 years old; therefore, they were interviewed along with their parents. Nine patients were followed by an asthma specialist (pulmonologist or allergist), 14 patients by family doctors or pediatricians, and one patient had no regular follow-up. Additional patient information is presented in Table 1. As case studies aim at achieving understanding of a phenomenon, we exceeded our initial target of 20 patients to maximize redundancy in patients’ reported barriers in this wide age spectrum.

**Table 1:** **Patients’ characteristics**

**Patient identification**	**Participant**	**Sex**	**Age category**	**Category of asthma duration (in years)**	**Followed by**		**Owns a written action plan**
					**Physician specialty**	**Workplace milieu**	
1	Patient	Female	20-30	20-24	Allergist	Non academic	No
2	Patient	Female	60-67	40-44	Pulmonologist	Academic	No
3	Parent + Patient	Male	6-12	Not reported	Pediatrician	Academic	Yes
4	Patient	Male	60-70	1-4	Family physician	Non academic	No
5	Parent + Patient	Male	6-12	5-9	Pediatrician	Academic	Yes
6	Patient	Male	13-18	Not reported	Family physician	Non academic	No
7	Patient	Female	13-18	< 1 year	Emergency physicians	Academic	No
8	Patient	Male	40-50	5-9	Pulmonologist	Non academic	No
9	Patient	Female	30-40	35-39	Pulmonologist	Non academic	No
10	Patient	Female	20-30	15-19	Family physician	Non academic	No
11	Patient	Female	50-60	10-14	Family physician	Non academic	No
12	Patient	Female	30-40	30-34	Family physician	Non academic	Yes
13	Parent + Patient	Male	6-12	5-9	Pediatrician	Non academic	No
14	Parent + Patient	Male	6-12	5-9	Pediatrician	Non academic	Not reported
15	Parent + Patient	Male	0-5	< 1 year	Family physician	Academic	No
16	Patient	Female	60-70	20-24	Pulmonologist	Academic	Yes
17	Patient	Female	70-80	1-4	Pulmonologist	Non academic	No
18	Patient	Male	30-40	1-4	Family physician	Non academic	No
19	Patient	Female	40-50	5-9	Pulmonologist	Academic	Yes
20	Parent + Patient	Male	6-12	1-4	Pediatrician	Academic	No
21	Patient	Female	70-80	20-24	Pulmonologist	Non academic	No
22	Patient	Female	20-30	20-24	Pulmonologist	Non academic	No
23	Patient	Female	20-30	25-29	Family physician	Non academic	No
24	Patient	Female	30-40	5-9	Family physician	Non academic	No

Inspired by the “Multi-level model of asthma disparities” [30] we classified reported themes [i.e., barriers and when available, matching facilitators] into three loci of responsibility, namely: patient, patient-physician interaction, and health care system. Themes were in turn sub-classified into a total of seven domains (i.e., cognition; motivation, attitude, and preference; practical implementation; parental support; communication; patient-physician relationship; and resources and services). A comprehensive list of barriers, facilitators, and exemplifying excerpts is available in Additional file 1: Table S1.

#### Patient-related locus

We included in this locus psycho-social barriers and facilitators to adherence, that is, those related to the patients’ personal development, behavior, and interaction with the family. Barriers and facilitators were further grouped into four domains: cognition; motivation, attitude, and preference; practical implementation; and parental support.

### Cognition

We classified within this domain barriers and matching facilitators related to patients’ beliefs, perceptions, fears, and knowledge. The *belief that their asthma is not serious* was reported by patients who refrained from taking their medication because they disregarded the importance of their symptoms and often did not recognize asthma as a chronic condition that needed to be controlled to prevent short-term events and long-term sequelae. *Fears of addiction or dependence to their medication* were raised by patients who expressed concerns of becoming dependent on medication. Similarly, some patients mentioned the *belief of decreasing effectiveness of the medication over time*, as they assumed that daily use of medication would lead to a need for increased dosage in the future. None of these three barriers were matched by any facilitator.

The *perception that medication should be used in response to symptoms*, and not on a regular basis, translated in patients’ intake of medication solely when experiencing symptoms, with discontinuation when symptoms abated. In contrast, the *perception that self-management should be used in anticipation of triggers* was a facilitator reported by patients who understood the disease as a chronic condition, accepted the need for long-term use of medication, and knew how to increase dosage of medications in anticipation of potential triggers that could result in an asthma flare-up/exacerbation.

Another barrier was *inadequate or limited knowledge about their medication*, reported by patients who were unsure about whether: they were taking the right medication, they were using the appropriate technique and dose, and/or the medication they were taking was compatible with medications taken for other conditions. In contrast, patients who reported *being knowledgeable about their medication* with regards to how to take it, how it worked, and what could be expected from its use, described this knowledge as a key facilitator to adhering to prescribed medications.

Patients also brought up two cognition-related barriers that were matched by the same facilitator. The *fear of adverse effects of medication* associated with use of an inhaled corticosteroid alone or in combination with a long-acting ß2-agonist and the *belief that the medication is not helpful or necessary,* justified from the patients’ perspective why they had stopped taking the asthma controller medication. A facilitator that counteracted these two barriers was the *perception of beneficial effects of medication,* which was often perceived as more important than their worries about the side effects of the asthma controller medication.

### Motivation, attitude, and preferences

We grouped, within this domain, four barriers and two facilitators. *Forgetfulness* about taking their medication was overcome by having *established routines for taking their medication*.

Patients suffering from a *lack of motivation* explained that it eroded over time, in part, because asthma was a chronic disease and they had to deal with it throughout their lives. Conversely, having a *proactive attitude* was a facilitator to adhering to long-term controller medication because it helped patients obtain the necessary information and resources needed to adequately deal with the disease.

Other barriers included a *preference for a non-pharmacological approach* discussed by patients who had a certain reticence towards taking traditional medications and reported developing parallel strategies to avoid taking medication, such as cleaning the nasal airways to improve breathing or relaxing in the presence of a flare-up. Some patients expressed a *preference for restriction of daily physical activity instead of taking medication,* preferring to avoid physical effort to avoid symptoms and thus decreasing the perceived need for asthma controller medication.

### Practical implementation

We classified within this domain themes related to the actual intake of medication. The *inconveniences of medication use* revolved around different features of the medication that the patients did not like (such as having to take the medication more than once a day), described as uncomfortable (e.g., the use of chambers), or that demanded additional actions (e.g., having to brush their teeth after its use). A facilitator was the *perception of medication as being patient-friendly* because of easiness and rapidity of medication intake.

Patients who brought up the *cost of medication* also discussed the existence of the Quebec *public drug plan* they benefited from, as a facilitator to adherence to long-term medication intake. They explained that, without this public drug plan that pays a substantial proportion of the cost of their medication, they would probably not be able to regularly take their medication.

Another facilitator patients discussed was *having a written action plan*. Patients explained that having a plan to monitor their symptoms and react in case of emergency reassured them. Also, some of the patients who did not receive a written action plan from their physician expressed positive opinions towards it and the intention of using it if they were given one.

### Parental support

Themes classified within this domain were solely brought up by patients’ parents and revolved around the collaboration among parents, as well as, the role of parental perception of the child’s disease. The *disagreements between parents about their child’s disease* led parents to approach their child’s treatment differently, a case that was more common in divorced parents. Instead, *agreement and partnership between parents,* was a facilitator beneficial for the child’s intake of daily controller treatment.

A second barrier was the *third-party perspective,* pertaining to the complexity of understanding the disease from a non-personal stance. The difficulty in understanding and interpreting a child’s symptomatology led parents to be unsure of the significance of the discomfort reported by their child. The gap in agreement on the interpretation of symptoms in the presence of asthma triggers between a child and his or her parents, usually led parents to delay therapy and observe the child for a certain period before administrating the needed rescue medication.

#### Patient-physician interaction locus

We included, in this locus, themes pertaining to the communication between the patient and the physician. We classified barriers and facilitators into two domains: communication and patient-physician relationship.

### Communication

The themes classified within this domain referred to the exchange of information between the patient and his or her physician. *Language limitations* referred to the fact that some instructions related to the disease and its management, including medication (e.g., dose, regimen), were given by physicians in a language (French or English) in which patients were not fluent. The lack of a clear understanding led patients to be reluctant to take the prescribed medication.

Medication adherence was also hindered by three communication barriers directly related to the diagnosis of asthma. The *misbelief or lack of a clear diagnosis* was reported by patients who, misguided by doubts concerning their diagnosis, refused to take long-term controller medication*.* According to them, a *clear diagnosis* of the disease resolved this barrier because once they were informed of their condition they could proceed accordingly. Other patients reported that their physicians had diagnosed them with asthma, but due to a *lack of formal or objective assessment of disease severity* (e.g., a respiratory function test), the severity of the disease remained unclear to them. Receiving a *formal or objective assessment of disease severity* was a clear facilitator; patients who took repeated lung function tests over time explained that they could more easily ascertain the impact of medication on their response to therapy. A third barrier was the *insufficient explanation of the condition and its management* in which patients hesitated to take medication when instructions on how to manage the disease were deficient. Conversely, *sufficient explanation of the condition and its management* improved reported adherence to asthma controller medication because patients felt knowledgeable enough to manage their asthma.

*Disagreement concerning the prescription and the management plan* was reported by patients who acknowledged that they needed to take long-term controller medication, but disagreed or were uncomfortable with either the prescribed dose, or the frequency, or the modality (e.g., inhalers vs. pills). Patients’ concerns and hesitations were dissipated when an *agreement on the prescription and the management plan* was reached as a result of negotiation with their physicians. *Adapting the information provided to the patients’ needs* was also a key facilitator. Patients reported the need for all explanations to be in lay language to accept the disease and to have a better understanding of the role of medications.

### Patient-physician relationship

A *poor patient-physician relationship* led patients to be less prone to taking the medication either because they did not like the physician’s attitude during the medical visit or felt that the prescribing physician was not sufficiently aware of their clinical history to make an optimal treatment decision. The latter was reported particularly by patients who, at a given point in time, had been followed by physicians working in an emergency department or at a walk-in clinic. Conversely, a *good patient-physician relationship*, where physicians expressed empathy towards the patients’ needs and took sufficient time to provide in-depth explanations and discuss concerns, encouraged patients to adhere to medication, even among those who were more reticent.

A second barrier was the *lack of a patient-centered approach*. Some patients felt that their physicians centered the asthma management strategy solely on prescribed medications, did not demonstrate empathy towards the patients’ situation, and/or appeared driven by the desire to minimize the visit duration. Conversely, a *patient-centered approach*, in which the physician and the patient shared a space to discuss the treatment, was reported as a key facilitator to long-term controller medication adherence because shared decision-making fostered the patients’ commitment towards therapy.

#### Health care system locus

Within this locus we classified barriers and facilitators related to the navigation of the health system. Only one domain, namely *resources and services,* was identified. Four barriers and four facilitators fell within this domain. Some patients referred to a *resistance to the medical context* and explained that sometimes, and for different reasons, they were reluctant to consult the medical system. These patients were less prone to follow medical recommendations.

The *lack of, or limited, health care resources* such as overloaded physicians, limited personnel, lack of specialists treating asthma, and long waiting lists, resulted in patients not getting adequate guidance and treatment in a timely fashion. In turn, *access to health care professionals, asthma education, and prescription renewal* notably facilitated adherence to long-term controller medication for asthma because being supervised and getting clear information through asthma clinics for example, made them feel reassured.

Some patients commented on the *lack of a structured follow-up plan*. This issue was brought up by patients who, in the past, did not have a formal follow-up. These patients were reticent to take a medication that could be associated with side effects, for an undetermined period of time, and without having medical supervision of the evolution of their disease. Conversely, a *structured follow-up by trained health care professionals* enhanced the patients’ intention to adhere to long-term controller medication intake because patients were reassured.

The *lack of, or poor, inter-professional communication* highlighted discordant or contradictory messages provided by different health care professionals that confused patients and lead them to proceeding as per their own inclinations, generally, against adhering to long-term controller medication for asthma. *Good inter-professional communication* was listed as a critical facilitator to ensure patients’ understanding and acceptation of both the disease and the treatment; and adherence to medication. Finally, some patients discussed one facilitator to medication adherence within this domain that matched no barriers: *improved treatments.* This facilitator was referred by patients who have lived with asthma for many years and explained that they found current treatments to be more effective that previous ones, which gave them an additional incentive to adhere to medication.

## Discussion

As our objective was to gain insight into factors perceived by patients as hindering or fostering their daily adherence to asthma medication and to develop a taxonomy of barriers and facilitators to support the development of interventions to increase patients’ adherence to daily long-term controller medication for asthma, we conducted face-to-face qualitative interviews with patients and parents of children with asthma. Among the barriers and facilitators that emerged in the analysis, some confirmed previous literature, others were rephrased to better depict participants’ voices as well as some underrepresented and unique findings. Based on an adapted version of the “multi-level model of asthma disparities,” [30] we organized barriers and facilitators according to three loci of responsibility, each with its correspondent domains, namely: (1) patient (cognition; motivation, attitudes and preferences; implementation; and parental support); (2) patient-physician interaction (communication and patient-physician relationship); and (3) health care system (resources and services). We proposed a taxonomy to support the development of knowledge translation interventions to increase patients’ adherence to long-term controller medication for asthma. Participants’ perspectives offered an interesting view of the interplay between facilitators and barriers related to patients’ reported behavior and intention regarding the long-term intake of prescribed inhaled corticosteroids, the cornerstone of asthma therapy.

Prioritizing the participants’ viewpoints allowed us to confirm and further enrich previously identified barriers to long-term controller medication for asthma [13-18] namely: distorted beliefs associated with the disease and the role, effectiveness and safety of medications; forgetfulness; lack of motivation; disagreement between parents concerning their child’s disease; poor patient-physician relationship; and issues related to health services and resources. Also, our findings led us to reformulate some barriers by closely reflecting the participants’ views. For example, we referred to patients’ “poor patient-physician relationship” rather than the more encompassing “dislike of the provider” [13] because these patients stressed the characteristics of the relationship, rather than an overall and imprecise dislike of the provider. We also identified new barriers pertaining to the patient locus (e.g., preference for restriction of daily activity instead of taking medication); at the patient-physician interaction locus (e.g., disagreement concerning the prescription and the management plan); and at the health care system locus (e.g., resistance to the medical context).

Our interest to identify patient-perceived facilitators to adhering to long-term controller medication for asthma underlied the fact that although it is important to understand patients’ perceived facilitators when developing knowledge translation interventions, this topic has been scarcely researched and reviewed. We confirmed several facilitators to long-term controller medication for asthma described in previous literature, such as having a written action plan and patients’ knowledge related to the medication and the disease [19,21]. In addition, we identified new items that serve to specify and enrich facilitators already discussed in previous research. For example, according to asthma educators interviewed by Chong and colleagues, [19] a child’s initial diagnosis of asthma may cause parental stress; however, over time, most parents gain confidence and appeared to cope with their child’s asthma fairly well. Based on our findings, we may speculate that the level of agreement and partnership between parents with regards to their child’s diagnosis and treatment, likely to evolve over the time, may contribute to modify parental confidence in dealing with their child’s disease.

We also identified certain facilitators that may enhance the translation of intention into sustained behavior, such as perceiving the beneficial effects of medication, having established routines for taking medication, and having a proactive attitude. Similarly, adapting the feedback to the patients’ need and involving the patient in the choice of medication and frequency of daily use, two facilitators identified in this study, represent specific instances of considering the patients’ preference and convenience when elaborating a treatment plan [17,19] Of note, the perception of medication as being patient-friendly, the availability of a public drug plan, and improved treatments were facilitators discussed by these patients, but not described in previous research. Notably, several facilitators were related to the interaction between patients and physicians such as agreement with the prescription and the management plan; a high-quality patient-physician relationship; and a patient-centered approach during the medical visit. These observations are consistent with prior reports identifying trust and good bi-directional communication as core constituents of a patient-physician relationship [32,33]. Our proposed taxonomy of facilitators, derived from patients’ report of adherence to long-term controller medication, is different from, and more exhaustive than our prior taxonomy of facilitators of physicians’ adherence to asthma guidelines in the emergency department; [22] yet, similar loci of responsibility were identified: physician, patient, and health care setting.

The proposed multi-level taxonomy carries two main implications concerning potential interventions. First, most facilitators were naturally paired with corresponding barriers, within a specific locus and domain. For instance, some barriers falling under the responsibility of the patient, such as forgetfulness, could be easily overcome by patients who established specific routines to take medication. Similarly, disagreements between parents about their child’s disease that lead to conflicts concerning the administration of medication, may be overcome by parental discussions leading to agreement and partnership concerning their child’s diagnosis and treatment. Second, although the proposed classification serves to guide the loci of responsibility, some barriers may be overcome by facilitators pertaining to a different locus or domain. For example, the belief that asthma is not serious, a cognition-related barrier classified within the patient locus, may be associated with the misbelief or lack of a clear diagnosis or a mis-communication at the patient-physician interaction locus and/or the lack of a structured medical follow-up attributed in part to the health care system. According to these findings, the quality of the patient-physician interaction, including negotiation concerning treatment goals and management plans, may be an effective way to address the multiple patient-related obstacles to adherence. Finally facilitators pertaining the health care system locus, such as the provision of drug insurance, free/low cost asthma education, and timely access to physicians, may have a positive impact on both the patient- and the patient-physician interaction loci.

Our observations of matched barriers and facilitators are supported by promising or successful patient-related interventions. For example, having experienced beneficial effects of the medication may be used as a probe in interventions using motivational interviewing or communication approaches, which consists of helping patients to identify their ambivalence concerning a given behavior [34]. Although there is preliminary evidence for a positive effect of motivational interviewing on medication adherence, [35] these results have to be considered cautiously due to small sample sizes and the pilot nature of some studies [36]. Patient-centered approaches or interventions, adapted to the specific needs and characteristics of patients, have been shown to be effective to address patient-related barriers and change patient behavior [37,38]. Furthermore, the rationale for a standardized ‘Educating the educators approach,’ endorsed by the Quebec Network for asthma education, the Canadian Network for Asthma Care, and other organizations to ensure the consistency of messages across health care asthma educators and providers, relies on the concordance of messages across professionals, a facilitator discussed by our patients [39]. We may speculate therefore that the success of a written self-management plan incorporated in a prescription, shown to improve medication adherence, probably facilitates a better communication between physicians, pharmacists, and patients, [21,40] a facilitator highlighted by patients.

In addition to the points discussed above, several patient-related barriers could be favorably influenced by the quality of the patient-physician interaction (e.g., patient-centered approach), strategies aiming at empowering patients’ self-management (e.g., medication reminders) or access to specific health care resources (e.g., asthma education, lung function testing); consequently, the approach to behavioral change should be guided by a comprehensive multi-level perspective [37,41-43].

With regards to generalizability, we recognize that several barriers identified in the previous literature were not mentioned by patients in our study, such as interference of life hassles, lack of social support, high levels of anxiety, and reliance on the belief of God [13,15,44]. While we cannot rule out the possibility that we did not achieve saturation, these barriers were possibly not reported due to the socio-cultural and contextual characteristics of our specific population. For example, stigmatization may indeed be of low concern to our patients. Similarly, few previously identified facilitators, such as parents’ positive view of asthma, [45] were not brought up by our patients. Also, although proven effective to improve adherence, [21] only five of our 24 patients reported having a written self-management plan. In addition, some barriers and facilitators identified in this study have been previously reported and would therefore appear to apply to different settings, we must acknowledge that their relative importance may be influenced by the local culture and health care system. Thus, we encourage researchers and health-care decision makers to identify key facilitators in their target population before implementing or testing a knowledge translation intervention.

This study has several strengths and limitations. We used a case study because this research strategy is an effective methodological research approach that results in a comprehensive and integrated knowledge [46-48]. The interview was flexible and allowed us to collect the necessary information to achieve our objective, respecting and following the participants’ logics. As some characteristics of asthma care (e.g., first vs. renewed prescription of long-term inhaled corticosteroids) and participants (e.g., asthma severity and control, delay since diagnosis of asthma, adherence to medication) were not documented, the relationship between barriers, facilitators, asthma control, and medication adherence could not be explored. With 18 patients over broad age spectrum and six parents interviewed, we identified one specific difference in the type of barriers and facilitators endorsed; this domain was named ‘parental support’ referring to issues only parents of children with asthma mentioned, such as disagreements between parents about their child’s disease’ and ‘third-party perspective.’ The diversity of participants’ age, roles (i.e., adult and adolescent patients, parents and children), health care provider specialty, and medical context brought a wide spectrum of perspectives that we specifically sought.

We did not designed the study to firmly identify differences in the endorsement of barriers and facilitators associated with specific patient characteristics (e.g., sex and role) and specific outcomes (e.g., asthma control or medication adherence); however, the proposed taxonomy will support future studies exploring endorsement variations across patients. While qualitative research does not aim at generalization, the consistency of reported barriers and facilitators that matched those identified in previous literature, emphasizes the validity of our findings. The close match between barriers and facilitators underlines the robustness of the proposed classification by domains and loci of responsibility. As our study was conducted in a setting with universal health care and subsidized drug plans, certain barriers or facilitators may not have been reported, thus affecting the generalizability of our findings to other health care contexts. We did not specifically record patients’ self-report of adherence or objectively documented adherence to long-term controller medication for asthma. Although facilitators identified in this study genuinely arise from discussions held with patients, there is usually a gap between intention and behavior, [49] such that the implementation of a certain facilitator may not systematically result in behavioral changes. Yet, several reported facilitators have been previously shown effective in improving adherence. Nevertheless, future research should both explore the value of untested facilitators using objective documentation of medication intake and validate the taxonomy we have developed.

The present study complements a similar parallel research endeavor seeking the perspective of physicians regarding barriers and facilitators to prescribing long-term controller medication for asthma [25]. Of interest, a large proportion of facilitators voiced by physicians were also identified by our participants, including patients’ attitude, physician communication skills, inter-professional management approach, and patients’ education concerning the disease and the medication, thus lending further support to the validity and applicability of our findings.

## Conclusions

While patients’ behavior falls within their own responsibility, the quality of interaction with the physician (e.g., shared decision making) and access to key health care resources (eg., lung function testing) and services (e.g., drug insurance) appear to play crucial roles in enhancing or impeding patients’ adherence to long-term use of inhaled corticosteroids, and importantly, to modify patients’ cognition, motivation, attitudes, and behaviors. Consequently, patients’ adherence to a treatment plan should be approached as a multi-faceted phenomenon. Anchored in previous research, we offer an enriched classification of barriers and facilitators to adherence to therapy, issued from the concordance between both concepts, that may enable the identification of population-specific solutions to improve adherence that could be formally evaluated subsequently. Although it deserves validation, the proposed taxonomy may be applicable to other populations and chronic conditions to plan intervention studies.

## Appendix A. Interview guide

*The goal of the present interview is to know more about your vision of asthma. We want to better understand, from for your perspective, what are the challenges related to the treatment, and especially to the self-management of asthma. We also want to explore the possible facilitators to face those challenges.*

### A. Introduction/Ice breaker

A.1. How did you learned that you (or your child) was asthmatic?

(*Explore whether the patient has been evaluated by his or her physician/s or whether he or she has just received a medication prescription without being formally diagnosed*)

A.2. Who follows your asthma? How did the/your physician/s and/or healthcare professionals describe asthma to you?

### A.3. According to you, what are the objectives to be attained during the treatment of asthma?

(*If it was not discussed by the patient, explore whether his or her asthma is considered mild or severe and what were the elements that supported the diagnosis*)

### B. Medication intake

B.1. What type of medication/s are you taking for your asthma? How do you take it? Do you follow the recommendations prescribed by your physician? What do you think of this medication?

(*If not mentioned by the patient, explore the use of a long-term controller medication and the meaning of “long-term”*)

B.2. Why do you take (or not) the medication in the way you have just described?

*(Explore different possible sources of influence, and among them, which one was the most important for the patient).*

B.3. (*If needed, and adjusted depending on the patients’ responses to B.1. and B.2.*)

What would you think/do if your doctor recommends to you to take long-term controller medication (i.e., inhaled steroids/corticosteroids)? *(Explore the perception of advantages and disadvantages associated to this type of prescription and if relevant, see what could help or prompt them to take it).*

### C. Consideration of medical recommendations

C.1. What do you do when your asthma worsens?

C.2. Why do you react this way when your symptoms worsen?

*(Explore different sources of influence, and if there are many, see which one was the most important)*

C.3. What helps you the most to manage periods of asthma aggravation? What hinders or

makes it more difficult?

C.4. (*If relevant and adjusted depending on the participant’s responses to C.1., C.2., and C.3.*)

What would you think/do if your physician recommends you to follow a written action plan to manage your asthma?

*(Explore the advantages and disadvantages of this type of prescription practice, and if relevant, see what could help or prompt them to follow a written action plan)*

### D. Patient-physician relationship

D.1. How would you describe your meetings with the physician/s and health care professionals that treat/s your asthma?

D.2. In your opinion, what is your role in the treatment of asthma? What is the physician’s role?

And what is the health care professionals’ role?

## Additional file

Additional file 1: Table S1. Patients’ excerpts exemplifying perceived barriers and facilitators to adherence to asthma medication.
