# Supplementary material for: Patients’ perspective of barriers and facilitators to taking long-term controller medication for asthma: a novel taxonomy
Source: BMC Pulm Med. 2015 Apr 25;15:42. doi: 10.1186/s12890-015-0044-9 (PMC4429418; doi:10.1186/s12890-015-0044-9)
Supplement: Additional file 1: Table S1. — Patients’ excerpts exemplifying perceived barriers and facilitators to adherence to asthma medication. [file 12890_2015_44_MOESM1_ESM.pdf]

Table S1

Patients' excerpts exemplifying perceived barriers and facilitators to adherence to asthma medication

Barriers	Facilitators
Patient-related locus	
Cognition domain	
• Belief that their asthma is not serious<br>*"Sometimes I have trouble to breathe, so I take the inhalers and after that, everything goes well. So I assume it is not that serious." (PT-04, a 61 years old male patient)*<br><br>• Fear of addiction or dependence to their medication<br>*"It scared me because my body, at a given point in time, won't learn how to breathe as it should and I will become dependent of that, I mean, my bronchial tubes will always need to be open with cortisone." (PT-24, a 33 years old female patient)*<br><br>• Belief of decreasing effectiveness of the medication over time<br>*"I took the medication for a long while and then I saw it was no longer efficacious." (PT-10, a 24 years old female patient)*<br><br>• Perception that medication should be used in response to symptoms<br>*"Yes, well, taking the medication is not the last, last, last*	• Perception that self-management should be used in anticipation of triggers<br>*"It's sure that when I exercise… I have to take my inhalers*

solution; I really wait until I feel somewhat uncomfortable. As such, I decided to decrease the amount of medication I should be taking." (PT-12, a 37 years old female patient)<br><br>• Inadequate or limited knowledge about their medication<br>*"I don't how to use this medication; I never know whether I have to turn it one or two times before breathing" (PT-09, a 37 years old female patient)*<br><br>• Fear for adverse effects of medication<br>*"No, I don't like taking the medication because it makes me shake like that (showing her hands) for two to three minutes" (PT-24, a 33 years old female patient)*<br><br>• Belief that the medication is not helpful or necessary<br>*"I am no longer taking the medication… I don't know what is it useful for and I see no changes!" (PT-11, a 57 years old female patient)*	*because I know the effort is going to be intense." (PT-01, a 29 years old female patient)*<br><br>• Being knowledgeable about their medication<br>*"I knew already how to take the medication. It's always the same way: I inspire, I expire and then, I press the button. So medication is really not a problem, I am used to it, I know how it works." (PT-06, a 18 years old male patient)*<br><br>• Perception of beneficial effects of medication<br>*"By the time he did the second breathing test, he was 5 years and it didn't raise any additional issues. I guess maybe the medication had helped to stabilize his condition, so I keep giving it to him." (The mother of PT-03, a 10 years old boy)*<br>*"I really need it! I feel a huge difference when I take the medication…I would say it has changed my quality of life in winter and I feel as well more self-confident." (PT-04, a 61 years old male patient)*
Motivation, attitude, and preference domain	
• Forgetfulness<br>*"I am lazy and I don't think of taking my medication. Technically, I know what this medication is good for, that it's good to take it regularly, and that is its long-term action that makes it efficacious. But it's because… at a given point… I am lazy, I think, I know I have to, but I don't do it… Conversely, I always have in mind I have to give my kids their medication, and the difference is that it's just for them!" (PT-24, a 33 years old female patient)*	• Established routines for taking their medication<br>*"For me, it's easy. I know I have to take my medication when I get up and when I go to bed. Taking my medication falls into my daily routine." (PT-04, a 61 years old male patient)*

• Lack of motivation<br>*"At a given point in time, we lose our motivation concerning the treatment. I am at this point now, I am not motivated…. So I gave up and I don't take the medication." (PT-11, a 57 years old female patient)*<br><br>• Preference for a non-pharmacological approach<br>*"I prefer something more natural… Probably, if the physicians do more research they can find out (PT-23, a 27 years old female patient)*<br><br>• Preference for restriction of daily physical activity instead of taking medication<br>*"We tried to sensitize him to avoid exercising the days we give him the inhalers. We tell him: 'Today, at the school backyard, you can't run because we gave you the medication. In order to try to regain your ordinary rhythm, you can play with your friends, but those days, try to be calm'. And it works!" (The mother of PT-03, a 10 year old boy)*	• Proactive attitude<br>*"I am the type of patient that pushes to have responses and says to the physician: "I won't leave your office until you explain to me what's going on." (PT-01, a 29 years old female patient)*
Practical implementation domain	

• Inconveniences of medication use<br>*“Always being forced to brush their teeth after using it” (PT-23, a 27 years old female patient)*<br>*“The medicine tasted nasty” (The mother of PT-20, a 9 year old boy)*<br>*“I hate the aero-chamber because it’s long to use and it’s difficult to transport” (PT-10, a 24 years old female patient)*<br><br>• Cost of medication<br>*“Medication is expensive! Every single time I go to the drug store to pick the medication it’s almost 200$ and that notably affects my budget… It’s sure that we are eventually reimbursed, but in any case, you have to pay it first!” (The mother of PT-03, a 10 year old boy)*	• Perception of medication as being patient-friendly<br>*“It’s going better… It is easy because when the powder is there, I pierce it and I inspire it.” (PT-08, a 41 years old male patient)*<br>*“The taste it’s not a problem; you brush your teeth and that’s it, it’s just that” (PT-18, a 33 years old male patient).*<br><br>• Public drug plan<br>*“Medication costs me nothing. I receive the social welfare, so my medication is covered. That’s why I could not take it otherwise” (PT-04, a 61 years old male patient)*<br><br>• Having a written action plan<br>*“Yes, it’s [the written action plan] easy to read and follow; really clear and helpful. We have everything in there: the indications and how to do it.” (The mother of PT-05, a 10 years old boy)*
Parental support domain	
• Disagreement between parents about their child’s disease<br>*“So the circle restarts, and it’s not nice neither for him (her son), nor for me, you know? We (parents) have discussed that a lot and I explained to him (the father) everything again and again: ‘I do this and that’ but he does not, so I frequently have to start his treatment from the very beginning, thus it’s even longer than expected, and at the end, I am not happy… And the problem is that he (the father) says he does not*	• Agreements and partnership between parents<br><br>*“This is the best we could do, because we have two younger children so one of us has to be at home looking after them. So he (her husband who is responsible for accompanying the child to medical appointments) received a lot of instructions and input from the doctors which he has transmitted to me second hand.” (The mother of PT-20, a 9 years old boy)*

<table>
<tr><td>believe he (the child) is allergic to anything, so in addition to that, he does not pay for the Epipen, you see?” (The mother of PT-03, a 10 years old boy)

• Third-party perspective
“But they (her parents) didn’t understand what was going on; you know? It’s easy to be lost when it’s not you the one who is ill, or the one who feels the symptoms, and you are even not in the body of the person who suffers.” (PT-01, a 29 year old woman who shared this passage from her childhood)</td><td></td></tr>
<tr><td colspan="2">Patient-Physician interaction locus</td></tr>
<tr><td colspan="2" align="center">Communication domain</td></tr>
<tr><td>• Language limitations
“My partner does not speak English and the physician who received us at the hospital ward did not speak French, so I was translating from English to French for him. However, I wanted him to understand what the physician was saying because he is the father and he needs to know how to give [name of the son] the medication.” (The mother of PT-13, a 12 years old boy)

• Misbelief or lack of a clear diagnosis
“(In the past) whenever he was having a crisis, I was going to different walk-in clinics. Physicians were prescribing him one medication, and then another one; but nobody told me, at that time, that he had asthma, so after a while, I was stopping the medication.” (The mother of PT-03, a 10 years</td><td>• Clear diagnosis
“Now he [her son] has been properly diagnosed and I know he has asthma, and even more, that I know what asthma is, so I make sure he takes his medication every day”. (The mother of PT-05, a 10 years old boy)</td></tr>
</table>

old boy)<br><br>• Lack of formal or objective assessment of disease severity<br>*I would say that I have light asthma, I guess, I don't know, because I never had a panic attack. If that is not the case, my asthma is just about cough, so I don't have a lot of problems to breathe and I do a lot of sport. Who knows? (PT-06, a 18 years old male patient)*	• Formal or objective assessment of disease severity<br><br>*"I have passed many, many different respiratory tests. That is why my medication has changed over the years…. The physicians following me made adjustments to medication based on both the tests I passed and my response to medication." (PT-01, a 29 years old female patient)*
• Insufficient explanation of the condition and its management<br>*"I find the physicians are expeditious… I find they don't take the time to take care of you." (PT-16, a 65 years old female patient)*	• Sufficient explanation of the condition and its management<br>*"So as I said, before I was just giving him the medication during his asthma crisis, but since he is followed by his new doctor, I stick to medication as prescribed because she made it clear to me the long-term benefits of following the prescription." (The mother of PT-05, a 10 years old boy)*
• Disagreement concerning the prescription and the management plan<br>*"They give you the inhaler automatically when the child has breathing difficulties… I don't know, but we could try something else, like shots." (The mother of PT-14, a 9 years old boy)*	• Agreement with the prescription and the management plan<br>*"If I want to be OK, then there is no other way than respecting the prescription. I asked the physician and he confirmed this to me. And I have to admit it, I am ok. So far, the medication has been doing what it is supposed to do." (PT-21, a 70 years old female patient)*<br><br>• Adapting the feedback to the patient's needs<br>*"You know, I am not a physician and he has the gift to explain me everything as many times as I need, in a way I can understand" (PT-01, a 29 years old female patient)*

Patient-physician relationship domain	
• Poor patient-physician relationship<br>*“This physician doesn’t know me and he is just giving me a prescription. How am I going to take this medication?” (PT-11, a 57 years old female patient)*<br>*“Well, my family doctor once gave a wrong pill and I told him: ‘… My God you know me for 30 years… Why did you give me that if you know I’m allergic to the penicillin?’” (PT-19, a 41 years old female patient)*<br><br>• Lack of patient-centered approach<br>*“Physicians check the blood pressure, examine the patient, and then prescribe something. But they (the physicians) don’t want to listen to you and you cannot talk to them while they write (laughs). They just want you to cross the door so you leave the place because there is another patient in the waiting room. It’s like that. And if you have another problem, they will say: ‘Just ask for another appointment.’ I know it, they did it to me very frequently, so you end up by being unmotivated, and the bottom line is that you don’t want to see physicians anymore!” (PT-11, a 57 years old female patient)*	• Good patient-physician relationship<br>*As it (the asthma crisis) normally happens at night, then at the emergency room there is a physician that works at night… He talks to me as if I were a young girl and it’s funny because he’s almost my older daughter’s age, he’s in his 40’s, but he talks to me as if I were my daughter’s age… so it’s always good to hear a friendly voice.” (PT-16, a 65 years old female patient)*<br><br>• Patient-centered approach<br>*“So it’s the two of us (she and her physician), together who find out what’s good for me, both at a medication and at an environmental level.” (PT-22, a 27 years old female patient)*
Health care system locus	
Resources and services domain	
• Resistance to medical context<br>*“And God knows I hate going to the hospital! I hate that! I*	

hate the emergency rooms, the physicians, I hate all related to the health system. If I go, it's just because I have no other choice" (PT-16, a 65 years old female patient)	
• Lack of a structured follow-up plan<br>*"I see him once in a while. Last time it was three years ago, so I would not call that a structured follow-up." (PT-18, a 33 years old male patient)*	• Structured follow-up by trained health care professionals<br>*"So it would be good if the patients could be followed in specialized clinics where they can receive appropriate treatment and follow-up." (PT-01, a 29 years old female patient)*
• Lack of, or poor, inter-professional communication<br>*"I know and I understand that there might be different positions concerning treatment, but I don't think the physicians who follow me are communicating well between them. They may give me, frequently, completely different advice, and as such, I end up by being completely lost." (PT-08, a 41 years old male patient)*	• Good inter-professional communication<br>*"In my case, even when the physician and the pharmacist always agree in the treatment, it is the pharmacist who usually goes a little bit further" (PT-11, a 57 years old female patient)*
• Lack of, or limited, health care resources<br>*"Now, physicians have lots of patients and therefore they don't have enough time to devote to each of us, as it was the case a while ago." (PT-18, a 33 years old male patient)*	• Access to health care professionals, asthma education and prescription renewal<br>*"Yes, I can go to see the physician and ask him if he can prescribe me my medicines… In general there's no problem, he can do that, following the pulmonologist's decisions." (PT-08, a 41 years old male patient)*
	• Improved treatments<br>*"So treatments have evolved and I find that marvelous. I find that is very good work and I hope this will go even better. It gives the taste to be on board, there you go!" (The mother of PT-15, a 2 years old boy)*
